# Transcription Factor E2F1 Knockout Promotes Mice White Adipose Tissue Browning Through Autophagy Inhibition

**DOI:** 10.3389/fphys.2021.748040

**Published:** 2021-11-08

**Authors:** Mingchen Xiong, Weijie Hu, Yufang Tan, Honghao Yu, Qi Zhang, Chongru Zhao, Yi Yi, Yichen Wang, Yiping Wu, Min Wu

**Affiliations:** Department of Plastic and Cosmetic Surgery, Tongji Hospital, Tongji Medical College, Huazhong University of Science and Technology, Wuhan, China

**Keywords:** E2F1 transcription factor, obesity, white adipose tissue (WAT), browning, autophagy

## Abstract

Obesity is associated with energy metabolic disturbance and is caused by long-term excessive energy storage in white adipose tissue (WAT). The WAT browning potentially reduces excessive energy accumulation, contributing an attractive target to combat obesity. As a pivotal regulator of cell growth, the transcription factor E2F1 activity dysregulation leads to metabolic complications. The regulatory effect and underlying mechanism of E2F1 knockout on WAT browning, have not been fully elucidated. To address this issue, in this study, the *in vivo* adipose morphology, mitochondria quantities, uncoupling protein 1 (UCP-1), autophagy-related genes in WAT of wild-type (WT) and E2F1^–/–^ mice were detected. Furthermore, we evaluated the UCP-1, and autophagy-related gene expression in WT and E2F1^–/–^ adipocyte *in vitro*. The results demonstrated that E2F1 knockout could increase mitochondria and UCP-1 expression in WAT through autophagy suppression in mice, thus promoting WAT browning. Besides, adipocytes lacking E2F1 showed upregulated UCP-1 and downregulated autophagy-related genes expression *in vitro*. These results verified that E2F1 knockout exerted effects on inducing mice WAT browning through autophagy inhibition *in vivo* and *in vitro*. These findings regarding the molecular mechanism of E2F1-modulated autophagy in controlling WAT plasticity, provide a novel insight into the functional network with the potential therapeutic application against obesity.

## Introduction

Obesity is a serious global public health problem and a preventable risk factor for hypertension, diabetes, breast cancer, and other cancers (Marie et al., 2015). Obesity is caused by the long-term excessive energy storage in WAT, including visceral adipose tissue and subcutaneous adipose tissue (Mori et al., 2014). Brown adipose tissue (BAT) possesses the ability to expend energy as heat, and is associated with UCP-1 expression. UCP-1 is located in the inner membrane of brown adipocytes mitochondria and uncouples the respiratory chain from oxidative phosphorylation through a proton conductance pathway (Villarroya et al., 2018). It is well-known that obesity is closely associated with adipose tissues, which play central roles in metabolic regulation (Kaisanlahti and Glumoff, 2019). Thus, further research on adipose physiological effects is needed to improve the understanding of metabolism and restore metabolic health.

When stimulated to a certain extent, brown adipocyte-like cells mainly appear in the subcutaneous storage of WAT, which is called “beige adipose tissue.” This metabolic process is called “WAT Browning” (Wang et al., 2015). Similar to BAT, beige adipose tissues present many small multilocular lipid droplets and densely packed mitochondria, with remarkable heat production ability through UCP-1-mediated mechanisms (Wang and Seale, 2016). There are several methods to activate thermogenic beige adipocytes within WAT during browning, including transcriptional and epigenetic regulation, lifestyle and environmental change, and endocrine factors and hormones secretion (Rabiee, 2020). Promoting the formation of beige adipocytes in WAT may potentially reduce the negative effects of excessive energy accumulation and improve overall metabolic health.

E2f Transcription Factor 1, the first member of the E2F transcription factor family, plays an important role in the regulation of the cell cycle, apoptosis, senescence, and DNA-damage response (Xiao et al., 2020). E2F1 has the ability to bind various gene promoter regions to regulate different biological functions (Wu et al., 2014; Ertosun et al., 2016). More importantly, E2F1 has been considered as a key regulator of metabolism both in normal and pathological conditions (Denechaud et al., 2017). Several metabolic tissues, including WAT, express higher E2F1 binding to the promoters of stress signaling genes in human obesity (Haim et al., 2017; Maixner et al., 2020). E2F1 repressed energy homeostasis and mitochondrial functions in muscle and brown adipose tissue, thus the mice lacking E2F1 were resistant to diet-induced obesity (Blanchet et al., 2013). The overall metabolic role of E2F1 in obesity suggests that E2F1 might play a significant role in gene regulation in adipose plasticity, thus needing further research.

Autophagy is a highly conserved cellular self-digestion pathway (Sun et al., 2018). Autophagosomes with bilayer membrane vesicles can trap the degraded substances and then fuse with the lysosome to degrade damaged cellular proteins and dysfunctional organelles (Wang et al., 2017, 2019). Amino acids and other degradation products produced by autophagy are recycled in the cytoplasm and help to maintain homeostasis (Yoshihara et al., 2014). Notably, E2F1 was proved to bind to regions encompassing the promoters of autophagy genes, thereby up-regulating the expression of microtubule-associated protein-1 light chain-3 (MAP1LC3), autophagy-related gene-1 (ATG1), and ATG5, and down-regulating the expression of sequestosome-1 (SQSTM1/p62) (Polager et al., 2008; Korah et al., 2016). In the setting of obesity, E2F1 expression was elevated in adipose tissue, which was relevant to the increased autophagy genes expression and the activated autophagy process (Haim et al., 2015). The correlation between E2F1, autophagy activity, and adipose tissue has great potential for balancing energy metabolism and controlling obesity.

Therefore, in this study, we aimed to investigate the regulatory effect of E2F1 knockout on WAT browning both *in vitro* and *in vivo*, as well as the potential autophagy-related mechanism. In general, we detected the adipose morphology, mitochondria quantities, UCP-1, autophagy-related genes in WT and E2F1^–/–^ mice *in vivo*. Furthermore, we evaluated UCP-1, and autophagy-related gene expression in WT and E2F1^–/–^ adipocytes *in vitro*. Lastly, our results confirm that E2F1-mediated autophagy is a novel pathway to regulate WAT browning, which is conducive to the development of therapeutic strategies for metabolic diseases such as obesity.

## Materials and Methods

### Animals

The E2F1^–/–^ mice were obtained from the Jaxson laboratory^1^ and were bred, maintained, and operated in the Animal Experimental Center of Tongji Hospital, Huazhong University of Science and Technology. In this study, the E2F1^–/–^ mice (male, 6–8 w) were compared with WT littermates of the same age and gender. All mice were allowed free access to food and water under controlled conditions (12/12 h light/dark cycle with humidity of 60 ± 5%, and a temperature of 22 ± 3°C). All surgical procedures were performed under anesthesia. The study was performed following guidelines on animal experimentation of the Ethical Committee of the Tongji Hospital.

### Isolation and Culture of Adipose Stem Cells (ADSCs)

The subcutaneous adipose tissues were extracted from the inguinal region of mice, and the superficial fascia and blood vessels were removed. Then, the adipose tissues were washed with phosphate-buffered saline (PBS) and minced into small pieces, followed by digestion using 0.15% type I Collagenase (Sigma, United States) under 37°C for 35 min. The Dulbecco’s modified Eagle’s medium (DMEM) (Gibco, United States) high glucose medium containing 20% fetal bovine serum (FBS) (Gibco, United States) was added to terminate the digestion, followed by filtration using a 75 μm screen mesh and centrifugation at 400 × *g* for 5 min. Lastly, the cells were added to the primary medium with 20% FBS and inoculated into a culture dish, and placed in an incubator with 5% CO_2_ at 37°C. After 24 h, the non-adherent cells were removed. These obtained ADSCs were passaged (1:3) using the culture medium containing 10% FBS every 2–3 days when they reached approximately 80–90% confluence.

### Adipogenic Differentiation and Oil Red O Staining

In a 6-well plate, 1 × 10^5^ ADSCs/cm^2^ were plated in complete media for 24 h. Differentiation was initiated by replacing complete media with adipogenic differentiation media, which contained 1 μM dexamethasone, 10 μg/μL insulin, 0.5 mM isobutylmethylxanthine, and 200 μM indomethacin. Cells were incubated for 21 days, and the media changed every 3 days. Murine 3T3-L1 preadipocyte cells were cultured and differentiated as previously described (Hwang and Lee, 2020). To assess the effect of autophagy on adipocyte differentiation, the autophagic inhibitor (3-MA), an inhibitor of phosphoinositide 3 kinase that specifically inhibits autophagosome formation at 5 mM (MCE, China), were added to the media before adipogenic differentiation.

The Oil Red O staining was used to evaluate adipogenic differentiation. The cells were rinsed with PBS and fixed with 4% formaldehyde, then were rinsed with distilled water, dehydrated with 60% isopropanol, stained with Oil Red O, and finally were observed under a light microscope (SDPTOP, China).

### Flow Cytometry Assay

The fourth-passage ADSCs were tested by flow cytometry analysis. Briefly, the adherent cells were harvested by trypsinization, centrifuged and washed with sterile PBS, and finally resuspended in PBS. For biomarker identification, cell suspensions were incubated with anti-CD29-APC, anti-CD90-FITC, anti-CD105-APC, anti-CD34-Alexa Fluor 647, and anti-CD44-PE⋅Cy^TM^ 7 antibodies (Becton Dickinson, United States). Finally, ADSCs were analyzed using a FACS Calibur cytometer (Becton Dickinson, United States) and Flow Jo VX software.

### Hematoxylin-Eosin (H&E) Staining

The adipose tissues were fixed in 4% formaldehyde and embedded in paraffin. Tissues were then cut into sections of 3 μm thickness with a microtome, deparaffinized, and stained with H&E. H&E images were taken with the microscope (SDPTOP, China) at 200 × and 400 × magnification. The adipocytes area quantification, represented by adipocyte diameter was performed in 6 fields of each preparation.

### Immunohistochemistry (IHC)

For IHC staining of UCP-1 expression level in WAT, formalin-fixed and paraffin-embedded adipose tissues were deparaffinized to prevent non-specific protein binding. Samples were then incubated with diluted primary anti-UCP-1 antibody overnight at 4°C. After the wash steps, the sections were incubated with secondary antibody for 2 h and were visualized by incubating with DAB substrate. The UCP-1 expressions were evaluated by high-power light microscopy examination. Images were taken with the microscope (SDPTOP, China) at 200 × and 400 × magnifications. The expression level of UCP-1 in adipose tissue was indicated with a stained positive index (integrated density/area).

### Transmission Electron Microscopy (TEM)

A small portion of WAT sections (1 mm^3^) were fixed with fresh TEM fixative at 4°C overnight and then washed the tissues using 0.1 M PB. Tissues were fixed with 1% OsO4 in 0.1 M PB (pH 7.4) for 2 h at room temperature. The tissues were dehydrated by a series of graded concentrations of alcohol and acetone and embedded in araldite. The resin blocks were cut to 60–80 nm thin and then stained with 2% uranium acetate and lead citrate, and observed under the TEM (Hitachi, Japan). The number of mitochondria in a region containing the complete view of an adipocyte was counted.

### Cell Transfection

The E2F1 siRNAs were purchased from Ribobio (Guangzhou, China). The sequences for the AGER siRNA used for the experiments were as follows: E2F1 siRNA1: 5′-GCAGAAACG GCGCATCTAT-3′; E2F1 siRNA2: 5′-GGGTGAGGGCATTAGA GAT-3′. ADSCs were plated in six-well plates and transient transfection using Lipofectamine 3000 Reagent (Invitrogen, United States) according to the manufacturer’s instructions.

### RNA Isolation, cDNA Synthesis, and qRT-PCR

The total RNA was isolated from the inguinal WAT of WT and E2F1^–/–^ mice using TRIzol (Takara, Japan), and 1 μg of RNA in a final reaction volume of 20 μL was then reversed-transcribed into complementary DNA (cDNA) using the 1st Strand cDNA Synthesis SuperMix (Yeason, China) according to the manufacturer’s instructions. qRT-PCR was performed using SYBR Green Master Mix (Yeason, China) and was detected with an ABI Q1 PCR System (Thermo Fisher Scientific, United States). Threshold cycles of primer probes were normalized to the housekeeping gene β-actin and translated to relative values. The following primer set sequences were used, LC3-II (forward 5′- TTATAGAGCGATACAAGGGGG AG-3′, reverse 5′-CGCCGTCTGATTATCTTGATGAG-3′), p62 (forward 5′-GAGGCACCCCGAAACATGG-3′, reverse 5′-ACTTATAGCGAGTTCCCACCA-3′), ATG5 (forward 5′-TG TGCTTCGAGATGTGTGGTT-3′, reverse 5′-ACCAACGTCA AATAGCTGACTC-3′), ATG7 (forward 5′-TCTGGGAAGCC ATAAAGTCAGG-3′, reverse 5′-GCGAAGGTCAGGAGCAGA A-3′), UCP-1 (forward 5′-GTGAACCCGACAACTTCCGA A-3′, reverse 5′-TGCCAGGCAAGCTGAAACTC-3′), E2F1 (forward 5′-GAGAAGTCACGCTATGAAACCTC-3′, reverse 5′-CCCAGTTCAGGTCAACGACAC-3′), and β-actin (forward 5′-GGCTGTATTCCCCTCCATCG-3′, reverse 5′-CCAGTTGG TAACAATGCCATGT-3′).

### Western Blot Analysis

Briefly, the protein concentration was quantified by the BCA method. The proteins were separated using 12% SDS-PAGE and transferred to a PVDF membrane. The membrane was blocked with 5% skim milk for 1.5 h and with 1:1000 dilutions of anti-LC3 antibody (Proteintech, United States), anti-p62 antibody (Proteintech, United States), anti-UCP-1 antibody (Proteintech, United States), anti-E2F1 antibody (ABclonal, China), and anti-β-actin antibody (ABclonal, China) overnight at 4°C. The membrane was then washed with TBST and incubated with a 1: 4000 diluted HRP-conjugated secondary antibody (ABclonal, China) for 1 h at 37°C. At last, the immunoreactive bands were detected by using an enhanced chemiluminescent (ECL) reagent kit (Yeason, China), and were scanned by ChemiDoc XRS^+^ imaging system (Bio-Rad, United States) and analyzed by Image Lab software. The analysis represented the ratio of the target protein expression relative to β-actin expression.

### Statistical Analysis

All experiments were performed with at least three replicates per group. Data are shown as the means ± standard deviation (means ± SD). Two treatment groups were compared by Student’s *t*-test. Statistical analysis was conducted using GraphPad Prism 9.0 software, and statistical significance was declared as ^∗^*p* < 0.05, ^∗∗^*p* < 0.01 and ^∗∗∗^*p* < 0.001, respectively.

## Results

### Effects of E2F1 Knockout on Body Weight and Inguinal Adipose Tissue Morphology in Mice

**FIGURE 1:**
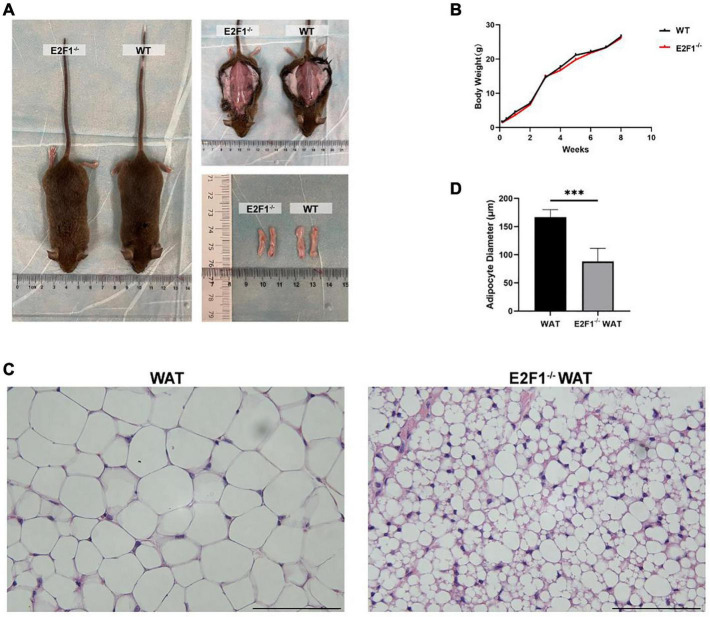
Effects of E2F1 knockout on body weight and inguinal adipose tissue morphology in mice. **(A)** Gross view of mice, *in situ* subcutaneous WAT, and *ex vivo* dissected WAT in WT and E2F1^–/–^ group. **(B)** The bodyweight curve of the WT and E2F1^–/–^ mice (*n* = 10) for 8 weeks. **(C)** Representative H&E staining images of WAT in WT and E2F1^–/–^ mice. Scale bar, 100 μm. Magnification, 400×. **(D)** Quantitative analysis of adipocyte diameter in WT and E2F1^–/–^ WAT. ^∗∗∗^*P* < 0.001.

Firstly, we intended to explore the impact of E2F1 knockout on the general morphology of mice and WAT. There was no obvious difference in the size and weight of subcutaneous inguinal WAT between the WT and E2F1^–/–^ groups (Figure 1A). Mice weight growth curve was measured weekly from the birthday, and there was no significant difference in weight between the two groups (Figure 1B). Interestingly, the microscopic observation revealed that the E2F1^–/–^ WAT exhibited substantially denser and smaller adipocytes (Figure 1C). The adipocyte diameter in the E2F1^–/–^ group was significantly shorter than that in the WT group (*P* < 0.001) (Figure 1D). These results certified that E2F1 knockout did not change the body and WAT weight, but reshaped the morphology of adipose. Thus, E2F1^–/–^ WAT might tend to be beige adipose morphology with multiple small lipid droplets.

### E2F1 Knockout Increased the Number of Mitochondria and the UCP-1 Expression in WAT *in vivo*

**FIGURE 2:**
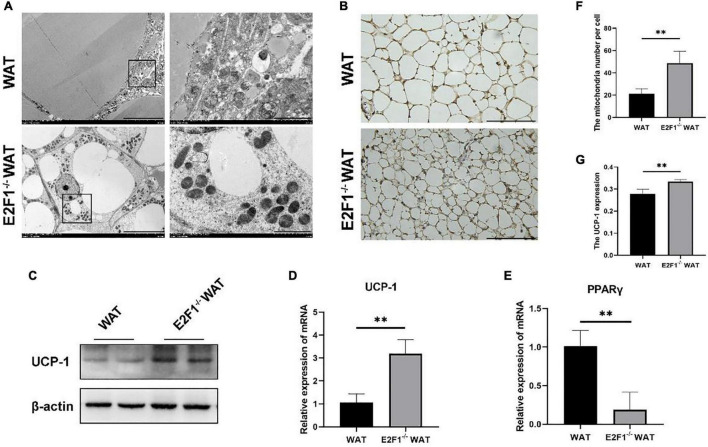
E2F1 knockout increased the mitochondrial number and the UCP-1 expression in WAT *in vivo*. **(A)** Representative TEM images of WAT ultrastructures in WT and E2F1^–/–^ group. Scale bar, 10 μm (left) and 2 μm (right). **(B)** Representative IHC pictures of UCP-1 protein (brown stain) in WAT of WT and E2F1^–/–^ mice. Scale bar, 100 μm. Magnification, 400×. **(C)** Representative Western Blot images of UCP-1 protein level in WT and E2F1^–/–^ WAT, *n* = 3. **(D)** The UCP-1 mRNA levels in WT and E2F1^–/–^ WAT in mice by qRT-PCR. **(E)** The PPARγ mRNA levels in WT and E2F1^–/–^ WAT in mice by qRT-PCR. **(F)** Quantitative analysis of mitochondrial number per cell in WT and E2F1^–/–^ WAT. **(G)** The expression level of UCP1 staining in WAT was indicated with a stained positive index (integrated density/area) in WT and E2F1^–/–^ WAT. ^∗^*P* < 0.05, ^∗∗^*P* < 0.01.

**FIGURE 3:**
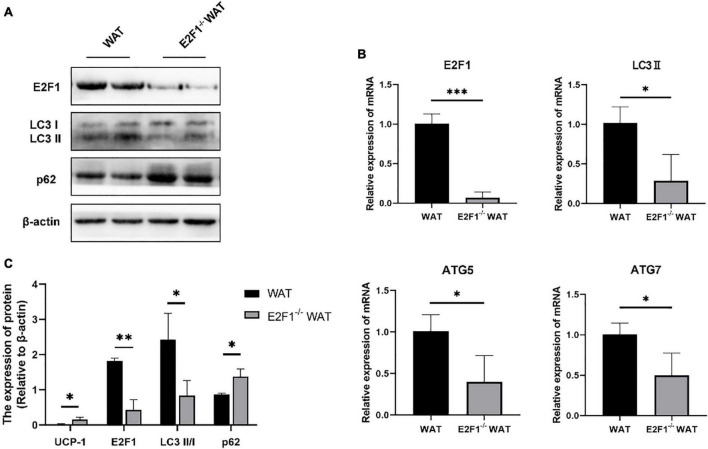
E2F1 knockout affected the expression of autophagy-related proteins in WAT in mice. **(A)** Representative Western Blot image of E2F1, LC3-II/I, and p62 protein levels in WT and E2F1^–/–^ WAT. **(B)** mRNA levels of E2F1, LC3-II, ATG5, and ATG7 genes in WT and E2F1^–/–^ WAT by qRT-PCR. **(C)** Quantitative analysis of the protein expressions relative to β-actin, *n* = 3. ^∗^*P* < 0.05, ^∗∗^*P* < 0.01, ^∗∗∗^*P* < 0.001.

Emerging evidence has confirmed that the increased number of mitochondria and high UCP-1 expression in WAT are strong indicators for browning. Consequently, we hypothesized that E2F1 knockout might induce WAT browning activity, which is related to mitochondria and UCP-1 in WAT. Intriguingly, E2F1^–/–^ WAT showed small intracellular lipid droplets and dramatically increased mitochondria, compared with the WT WAT (*P* < 0.01) (Figures 2A,F). In the WT group, the presence of multiple degraded vesicles was consistent with the structure of autophagosomes and autophagolysosomes, which suggested that autophagy activity might occur. The UCP-1 of IHC staining demonstrated that there were more UCP-1 expression and beige cells formation in the E2F1^–/–^ group (*P* < 0.01) (Figures 2B,G). Moreover, the Western Blot analysis showed that the expression of UCP-1 protein was significantly increased in the E2F1^–/–^ WAT (*P* < 0.05) (Figures 2C, 3C). Similarly, the qRT-PCR results consistently confirmed that E2F1^–/–^ WAT possessed higher mRNA expression of UCP-1 than WT WAT (*P* < 0.05) (Figure 2D). In addition, to validate the adipogenic gene expression during WAT browning, the PPARγ mRNA expression in the E2F1^–/–^ WAT group was detected, which exhibited a downward trend compared with the WT group (Figure 2E). These data indicated that E2F1 knockout induced WAT into beige adipose tissue by increasing mitochondrial number and expressing highly UCP-1 both on mRNA and protein levels.

### E2F1 Knockout Affected the Expression of Autophagy-Related Proteins in WAT *in vivo*

Since the autophagy-related autophagosomes expression was decreased in E2F1^–/–^ WAT, we further explored the underlying autophagy mechanism of E2F1 in regulating WAT browning. The E2F1^–/–^ group demonstrated the protein expressions of E2F1 and LC3-II/I were significantly decreased, and p62 was increased inversely (*P* < 0.05, *P* < 0.01) (Figures 3A,C). Compared with the WT group, the reduced mRNA expressions of E2F1, LC3-II, ATG5, and ATG7 in E2F1^–/–^ WAT were further confirmed (*P* < 0.05, *P* < 0.01) (Figure 3B). The results suggested that autophagy participated in the intracellular beige adipogenesis, while E2F1^–/–^ WAT exhibited lower autophagy gene expression than WT WAT.

### E2F1 Knockout Increased UCP-1 Expression in Adipocytes *in vitro*

**FIGURE 4:**
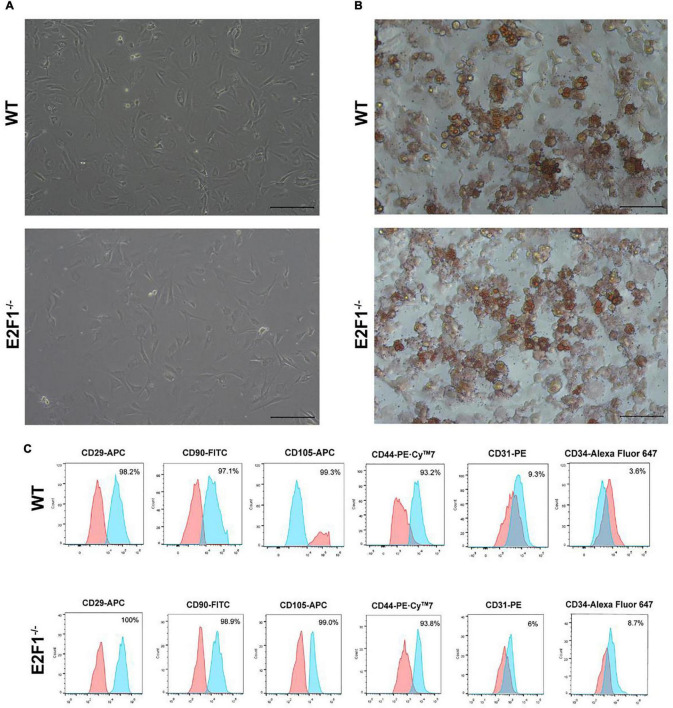
Characterization of mice ADSCs. **(A)** Representative images of WT and E2F1^–/–^ ADSCs (P3). Scale bar, 100 μm. **(B)** Adipogenic differentiation was analyzed by Oil Red O staining. Scale bar, 100 μm. **(C)** Flow cytometry of phenotypic markers of ADSCs. The WT and E2F1^–/–^ ADSCs were positive for CD29, CD90, CD105, and CD44, and negative for CD31 and CD34.

**FIGURE 5:**
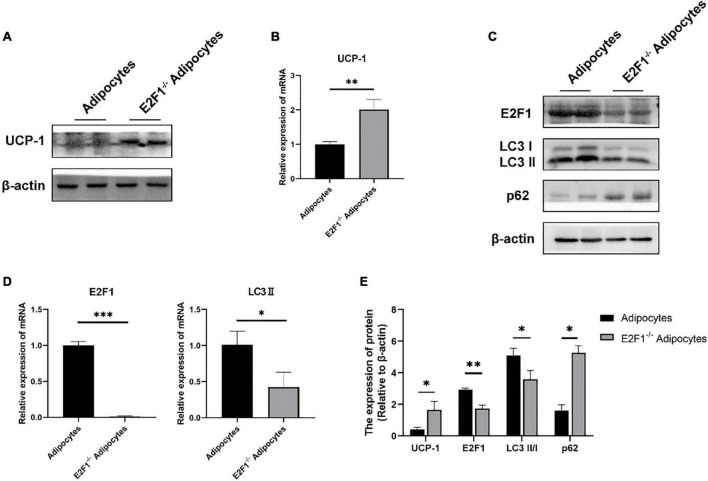
E2F1 knockout increased UCP-1 expression and reduced autophagy gene expressions in adipocytes *in vitro*. **(A)** Representative Western Blot image showing the level of UCP-1 protein in adipocytes and E2F1^–/–^ adipocytes. **(B)** qRT-PCR analyses of UCP-1 expression at the mRNA level in adipocytes and E2F1^–/–^ adipocytes. **(C)** Representative Western Blot image of E2F1, LC3-II/I, and p62 protein levels in adipocytes and E2F1^–/–^ adipocytes. **(D)** The qRT-PCR analyses of E2F1, and LC3-II expressions at the mRNA level in adipocytes and E2F1^–/–^ adipocytes. **(E)** Quantitative analysis of the protein expressions relative to β-actin, *n* = 3. ^∗^*P* < 0.05, ^∗∗^*P* < 0.01, ^∗∗∗^*P* < 0.001.

The cultured WT and E2F1^–/–^ ADSCs both presented a typical fibroblast-like morphology, which was similar to previous studies (Figure 4A). Flow cytometry analysis also verified the surface markers of obtained ADSCs, by staining positively for CD29, CD90, CD105, and CD44, and negatively for CD31 and CD34 (Figure 4C). The Oil Red O staining images indicated the presence of oil droplet formation, suggesting the acquisition of mature adipocytes *in vitro* (Figure 4B). To demonstrate the role of E2F1 in beige adipocyte modulation *in vitro*, the UCP-1 protein, and mRNA expression were analyzed. The Western Blot analysis testified that E2F1^–/–^ adipocytes possessed significantly high expression of UCP-1 (*P* < 0.05) (Figures 5A,E). The qRT-PCR results further proved the higher mRNA expression of UCP-1 in the E2F1^–/–^ group than WT group *in vitro* (*P* < 0.01) (Figure 5B). Thus, E2F1 knockout could increase the UCP-1 expression in adipocytes, which was necessary to the white-to-beige process.

### E2F1 Knockout Reduced Autophagy Gene Expression in Adipocytes *in vitro*

The autophagy inhibition was proved to occur in the E2F1^–/–^ WAT *in vivo*, thus participating in the WAT browning remodeling. To confirm that the role of E2F1 in beige adipocytes regulation depends on autophagy *in vitro*, the autophagy-related genes were detected. At the protein level, the LC3-II/I expression was decreased, and the p62 expression was increased in the E2F1^–/–^ adipocytes group, compared with the WT adipocyte group (*P* < 0.05, *P* < 0.01) (Figures 5C,E). Also, the reduced mRNA expression of LC3-II and the induced mRNA expression of p62 were further verified in E2F1^–/–^ adipocytes (*P* < 0.05, *P* < 0.01) (Figure 5D). The results proved that E2F1 could be involved in the upregulation of autophagy-related genes in adipocytes *in vitro*, and E2F1 knockout inhibited autophagy activity to promote WAT browning.

### Autophagy Inhibition Increased the UCP-1 Expression, Without Reduced E2F1 Expression in Adipocytes *in vitro*

**FIGURE 6:**
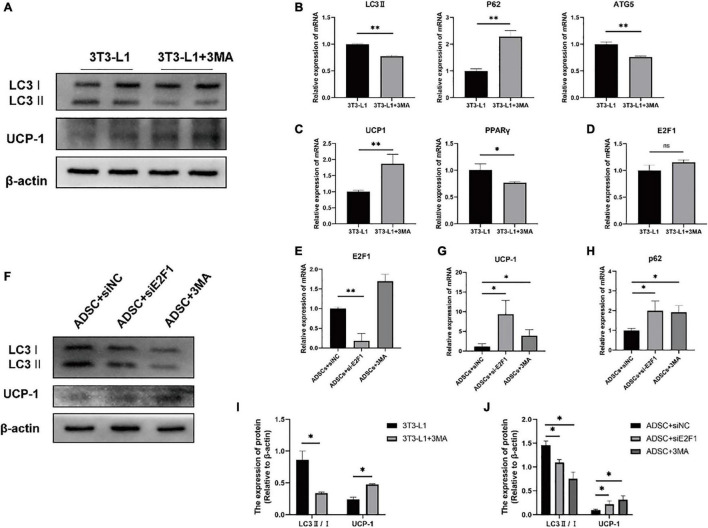
Autophagy inhibition increased the UCP-1 expression, without reduced E2F1 expression in adipocytes *in vitro*. **(A)** Representative Western Blot image showing the level of LC3-II/I and UCP-1 protein in 3T3-L1 adipocytes and 3T3-L1 + 3MA adipocytes. **(B–D)** qRT-PCR analyses of LC3-II, p62, ATG5, UCP-1, PPARγ, and E2F1 expression at the mRNA level in 3T3-L1 adipocytes and 3T3-L1 + 3MA adipocytes. **(E)** The qRT-PCR analysis of E2F1 expression at the mRNA level in ADSCs + siNC group, ADSCs + E2F1 siRNA group, and ADSCs + 3MA group. **(F)** Representative Western Blot image of LC3-II/I, and UCP-1 protein levels in ADSCs + siNC group, ADSCs + E2F1 siRNA group, and ADSCs + 3MA group. **(G,H)** The qRT-PCR analyses of p62, and UCP-1 expressions at the mRNA level in ADSCs + siNC group, ADSCs + E2F1 siRNA group, and ADSCs + 3MA group. **(I)** Quantitative analysis of the LC3-II/I and UCP-1 protein expressions relative to β-actin in 3T3-L1 adipocytes and 3T3-L1 + 3MA adipocytes, *n* = 3. **(J)** Quantitative analysis of the LC3-II/I and UCP-1 protein expressions relative to β-actin in ADSCs + siNC group, ADSCs + E2F1 siRNA group, and ADSCs + 3MA group, *n* = 3. ^∗^*P* < 0.05, ^∗∗^*P* < 0.01.

Firstly, we used the 3T3-L1 cell line as an *in vitro* model to generate adipocyte-like cells. To figure out whether the reduced autophagy could affect the UCP-1 and E2F1 expression, the 3T3-L1 adipocytes were treated with autophagy inhibitor 3-MA at the early differentiation stage. After adipogenic induction, the inhibition of autophagy using 3MA resulted in lower autophagy activity. The LC3-II and ATG5 mRNA expressions were relatively reduced compared to the normal cultured 3T3-L1 cells (Figures 6A,B,I). The p62 mRNA expression was increased in the 3T3-L1 + 3MA group (Figure 6B). Furthermore, the 3MA-treated 3T3-L1 adipocytes also expressed higher UCP-1 on mRNA and protein levels, and lower PPARγ mRNA expression than the 3T3-L1 adipocytes, which demonstrated that the autophagy inhibition leads to reduced lipogenesis and enhanced WAT browning transition (Figures 6A,C,I). Importantly, there was no significant difference in E2F1 expression between the 3T3-L1 + 3MA group and 3T3-L1 group (Figure 6D). It was indicated that the autophagy inhibition in the WAT could be induced by E2F1 knockout, leading to the increased mitochondria and UCP1 expression in the browning WAT.

Next, three groups were designed to examine the effect of E2F1 on autophagy inhibition and WAT browning, including ADSCs + 3MA group, ADSCs + E2F1 siRNA group, and ADSCs + siNC group. After ADSC adipogenic differentiation, the expression levels of E2F1 mRNA were reduced by si-E2F1 in ADSCs (Figure 6E). The 3MA addition did not lead to a decrease of E2F1 mRNA expression in the WT ADSCs + 3MA group. The Western Blot analysis proved that the LC3-II/I protein expression indeed was reduced in both ADSCs + 3MA group and ADSCs + E2F1 siRNA group (Figures 6F,J). The ADSCs treated with E2F1 siRNA mimicked the effect of autophagy inhibitor to increase the UCP-1 expression at protein and mRNA level (Figures 6F,G,J). In addition, the p62 mRNA expression was relatively induced in ADSCs + 3MA group and ADSCs + E2F1 siRNA group, compared to the ADSCs + siNC group (Figure 6H). These results further confirmed that E2F1 deficiency could be involved in UCP-1 expression via autophagy inhibition in WAT and adipocytes.

## Discussion

Obesity is caused by a metabolic disturbance between energy intake and energy expenditure. Brown and beige fats specialize in adaptive thermogenesis, expend energy as heat by decoupling mitochondrial respiratory chains and ATP synthesis, and play an increasingly important role in glucose homeostasis, insulin sensitivity, and lipid metabolism (Kaisanlahti and Glumoff, 2019). E2F1 is a widely recognized cell cycle regulatory transcription factor, and its role in human obesity is being emphasized (Haim et al., 2015). Imbalanced E2F1 activity leads to metabolic complications associated with obesity. In addition, the regulation of autophagy activity adapted to individual metabolism is another important factor in adipose tissue metabolism and function balance (Ro et al., 2019). Autophagy inhibition in the early stage could alter WAT characteristics into a “browning” state and improves glucose tolerance at later stages (Ghosh et al., 2018). In this study, we utilized the WT and E2F1^–/–^ mice to investigate the possible role and E2F1-modulated autophagy mechanism.

In the present study, primarily, there were no differences in body sizes, weights, the *in situ* subcutaneous WAT, and the *ex vivo* dissected WAT. Interestingly, Choi et al. reported that HFD-fed and ob/ob mice exhibited more expression levels of mRNA, including fibroblast growth factor receptor 1, cyclin D, and E2F1, compared to those of the normal diet-fed and lean control mice, respectively (Choi et al., 2016). Thus, the E2F1 knockout did not change the bodyweight, which might attribute to that we did not establish the obesity settings in mice. Besides, H&E staining revealed significantly smaller adipocytes size in the E2F1^–/–^ WAT compared with WT WAT, which presented “beige” adipose-like morphology. As Xue et al. reported that after exposure to cold temperatures, subcutaneous WAT of mice possessed relatively large, round, and condensed mitochondria with numerous transverse cristae surrounding smaller lipid droplets, indicating browning of WAT (Xue et al., 2018). Here, E2F1^–/–^ WAT showed smaller intracellular lipid droplets and more mitochondria quantities than WT WAT, revealing the beige adipose shape. High expression of UCP-1 is one of the important characteristics of WAT browning (Devlin, 2015). Blanchet et al. illustrated that E2F1^–/–^ BAT showed increased expression in the mitochondrial respiratory chain and uncoupling respiration (UCP-1, 2), compared to the WT BAT (Blanchet et al., 2013). In this study, the highly UCP-1 expression and beige cells formation were observed in E2F1^–/–^ WAT *in vivo* and E2F1^–/–^ adipocytes *in vitro*. These results demonstrated that E2F1 knockout promoted WAT browning through increasing UCP-1 expression and mitochondria, which might lead to energy expenditure in a thermogenic manner.

The C1q/TNF-related protein 5 (CTRP5) was reported as a novel adipokine that was significantly down-regulated in subcutaneous WAT of mice after being exposed to cold temperatures (Rao et al., 2019). The overexpressing CTRP5 elevated the autophagy level and suppressed UCP-1 expression, whereas the autophagy inhibitor could rescue the suppression. E2F1 acted as a key transcription factor and its knockout induced WAT browning along with the decrease in autophagy activity. We observed that the autophagosomes occurred with cytoplasm-like substances enclosed by a multilayered membrane in WT WAT under TEM. These results were similar to their counterparts observed by Kim et al. (2018). Besides, autophagolysosomes with monolayered structures also could be seen in the WT group. Instead, there were nearly no autophagosomes and autophagolysosomes appearing in E2F1^–/–^ WAT. The LC3 in the cytosolic form (LC3-I) was conjugated to phosphatidylethanolamine to form LC3-II, and then recruited to autophagosomal membranes. Hence, LC3-II/I is used as a key marker of autophagy (Tao et al., 2016). As an autophagic adapter, p62 interacting with LC3 was determined as another indicator of autophagy. Maixner et al. summarized that promotor binding, promoter activity, and autophagic flux measurement results identified the capacity of E2F1 to transcriptionally regulate autophagy genes and to activate autophagy in adipose tissue and adipocytes (Maixner et al., 2016). More interestingly, in the E2F1^–/–^ group, the LC3-II/I at the protein level was significantly decreased, indicating the reduction of autophagosomes. Consequently, the expression of mitophagy receptor p62 was increased due to the blockage of autophagy.

Autophagy has been confirmed as a key regulator involved in adipose tissue physiology, especially in WAT and BAT adipogenesis (Zhang et al., 2012). Mitophagy is an important type of autophagy that selectively removes excess or damaged mitochondria, positively regulating the white adipogenesis and negatively regulating the beige and brown adipogenesis (Altshuler-keylin et al., 2017). It is well-recognized that WAT maintains more autophagy activity to keep a lower number of mitochondria, whereas BAT and beige adipose tissue possessed higher mitochondrial biogenesis and lower mitophagy (Ferhat et al., 2019). The autophagy and mitophagy occurred dynamically depending on browning status. For instance, Cairó et al. (2016) demonstrated that autophagy inhibition in brown adipocytes resulted in the increase of UCP-1 protein and uncoupled respiration, suggesting that autophagy inhibition activated thermogenesis and was part of the adaptive mechanism of brown adipocytes. Moreover, in the course of transition from white to beige adipose tissue, the inhibition of mitophagy could help to maintain higher mitochondrial content for the beige adipose tissue remodeling. Singh et al. found that Atg7 knockout mice showed a decreased white adipose mass and enhanced insulin sensitivity (Singh et al., 2009). Besides, the defective autophagy in WAT promoted a phenotype with reduced WAT mass and increased browning adipocyte features. In the study of Armani et al., they also verified that mineralocorticoid receptor antagonism markedly reduced the autophagic rate both in murine preadipocytes *in vitro* and WAT depots *in vivo*, with a concomitant increase in UCP-1 protein expression (Armani et al., 2014). In keeping with the fact that our study also E2F1^–/–^ group *in vivo* and *in vitro* possessed decreased autophagy-related LC3-II, ATG5, ATG7 mRNA expression, and certainly increased p62 mRNA expression. It further proved that E2F1 knockout could alleviate the autophagy activity in WAT of mice. In our study, the reasons for E2F1-involved autophagy or mitophagy inhibition in WAT browning were not fully examined, but the observed enhanced mitochondrial content suggested a failure to clear mitochondria or impaired mitophagy, indicating that the thermogenic program inversely correlates with the autophagy program. It speculated that mitochondrial autophagy activity might be inhibited after E2F1 knockout in the WAT, contributing to an increase in the mitochondria number and higher mitochondria function. Thus, E2F1 knockout in WAT repressed the autophagy to up-regulate UCP-1 expression and promote WAT browning, which was expected to be used for fat burning to dissipate extra energy and obesity prevention.

## Conclusion

**FIGURE 7:**
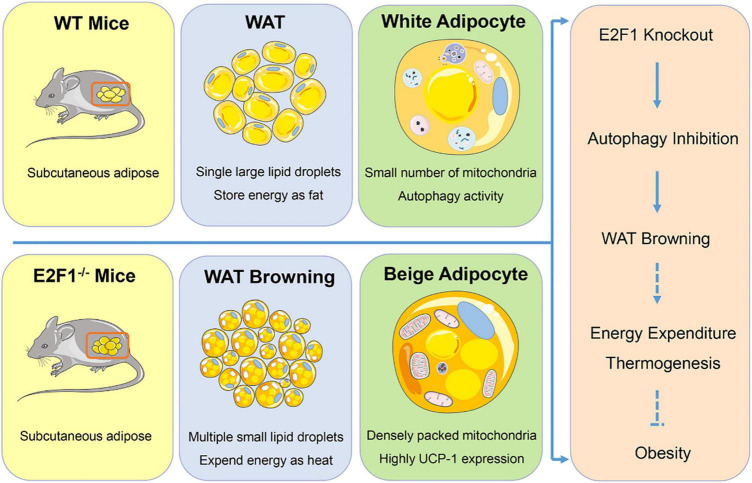
Graphical summary. E2F1^–/–^ WAT exhibited multiple small lipid droplets, densely packed mitochondria, and highly UCP-1 expression, indicating the browning form. Mechanically, E2F1 knockout suppressed the autophagy activity to promote WAT browning in mice, which was beneficial to expend energy in thermogenic manner for combating obesity.

In summary, our findings provided evidence that E2F1 knockout induced WAT and adipocyte browning through reducing autophagy activity both *in vivo* and *in vitro* (Figure 7). E2F1 could serve as a potential target to trigger mitochondrial activation in WAT and to promote WAT browning, which is conducive to increased energy expenditure and subsequent weight loss. The involvement of E2F1 in autophagy may be the entry point affecting WAT browning, providing a novel explanation for the autophagy regulation by transcription factors to determine energy metabolism balance and obesity control. On the other hand, some issues still should be addressed in further study Firstly, the supervised basic body temperature and basal metabolic rate are evidence for beige and brown adipose thermogenesis, which was not acquired in the results. Secondly, cold exposure or β-adrenergic stimulation is necessary to activate WT and E2F1^–/–^ WAT, contributing to more pronounced phenotypic and functional changes. Then, it will be of value to explore the role of E2F1 overexpression in E2F1^–/–^ adipocyte, which can be further confirmed E2F1 regulation by the rescue experiments. Lastly, the actual function of E2F1 needs to be explored in mice with a high-fat diet under obesity settings.

## Data Availability Statement

The original contributions presented in the study are included in the article/supplementary material, further inquiries can be directed to the corresponding author/s.

## Ethics Statement

The animal study was reviewed and approved by Animal Experimental Center of Tongji Hospital, Huazhong University of Science and Technology.

## Author Contributions

MX, WH, YT, and HY performed the experiment. MX and WH wrote the manuscript and finished the result analysis. QZ, YWu, and MW conceived the project and revised the manuscript. CZ, YWa, and YY edited the manuscript. All authors reviewed the manuscript and all approved of the final version.

## Conflict of Interest

The authors declare that the research was conducted in the absence of any commercial or financial relationships that could be construed as a potential conflict of interest.

## Publisher’s Note

All claims expressed in this article are solely those of the authors and do not necessarily represent those of their affiliated organizations, or those of the publisher, the editors and the reviewers. Any product that may be evaluated in this article, or claim that may be made by its manufacturer, is not guaranteed or endorsed by the publisher.

## Footnotes

1
http://jaxmice.jax.org/strain/002785.html

## Abbreviations

WAT: White Adipose Tissue
BAT: Brown Adipose Tissues
UCP-1: Uncoupling Protein-1
E2F1: E2f Transcription Factor 1
MAP1LC3: Microtubule-Associated Protein-1 Light Chain-3
SQSTM1/p62: Sequestosome-1
ATG: Autophagy-Related Gene
WT: Wild-Type
ADSCS: Adipose Stem Cells
FBS: Fetal Bovine Serum
PBS: Phosphate-Buffered Saline
H&E: Hematoxylin-Eosin
IHC: Immunohistochemistry
TEM: Transmission Electron Microscopy
CTRP5: C1q/TNF-related protein 5.
